# A novel green approach for reactive printing of cotton/cellulosic regenerated blended fabrics using trisodium nitrilotriacetate

**DOI:** 10.1038/s41598-024-75633-5

**Published:** 2024-10-23

**Authors:** Abdalla A Mousa, Alsaid A Almetwally, Sahar H Nassar, Nahed S Ahmed, Hesham M Fahmy, Reda M El-Shishtawy

**Affiliations:** 1 Dyeing, Printing and Dye Intermediates Department, Textile Research and Technology Institute, National Research Centre, 33 EL Buhouth St, Dokki, 12622 Giza Egypt; 2 Textile Engineering Department, Textile Research and Technology Institute, National Research Centre, Dokki, 33 EL Buhouth St, Dokki, 12622 Giza Egypt; 3 Pre-Treatment and Finishing of Cellulosic Fabric Department, Textile Research and Technology Institute, National Research Centre, Dokki, 33 EL Buhouth St, Dokki, 12622 Giza Egypt

**Keywords:** Environmental printing processes. Regenerated cellulosic fabrics, Blends printing, Trisodium nitrilotriacetate, Reactive printing and reactive dyes, Environmental sciences, Chemistry

## Abstract

Owing to their comfort, handle, and aesthetic characteristics, cotton fabrics will always be the first and primary choice for clothing and apparel. In recent years, regenerated cellulosic fabrics like bamboo, Tencel, and modal fabrics have had many natural advantages. Fabrics based on blending cotton fibres with regenerated cellulosic fibres are considered promising products in textile industry sectors. Use of urea poses ecological problems associated with the high nitrogen content of the printing effluent. Therefore, urea reduction or elimination in reactive dye print pastes is of ecological interest. We report the use of trisodium nitrilotriacetate as a complete substitution of urea and alkali in the conventional reactive printing of cotton/cellulosic regenerated blended fabrics. CI Reactive Black 5 was selected for the present study. Three different print pastes containing urea/alkali, trisodium nitrilotriacetate/alkali and trisodium nitrilotriacetate without alkali were thoroughly investigated. Different factors that may affect the printability of cotton/cellulosic regenerated blended fabrics, such as the concentrations of dye, trisodium nitrilotriacetate, urea, absence or presence of alkali and steaming time in the prints obtained, were evaluated concerning colour strength, dye fixation, dye penetration, levelling, colure, and fastness properties. All printed fabrics using three print pastes obtained excellent to good fastness. The results proved the viability of using TNA as an environmentally friendly approach for urea/alkali-free printing of cellulosics with reactive dyes.

## Introduction

 Fiber blending can be defined as mixing two or more different fibers or the same fibers with other characteristics to produce a single yarn, referred to as blended yarn^1^. Various functional fibers offering different and highly available properties such as high stretch, water repellence, flame retardant, wicking, and electrical conductivity have recently emerged. The lifecycle of Tencel fabrics, as an example of such functional fibers has minimal environmental impact than synthetic fabrics (e.g. polyester, nylon, and acrylic) and natural fabrics such as cotton fabrics, which require significant areas of land, irrigation, pesticides and fertilizers to grow^2^. Blending the functional fibers with traditional ones, whether man-made or natural, will open new opportunities for developing textile end products^3^. In this regard, the dyeing of modal/cotton blended fabrics with reactive and sulphur dyes exhibited very good fastness properties and could be best utilized for different varieties of garments and textile products^4^. Tencel blended fabrics, namely Tencel/silk and Tencel/polyester fabrics are dyed with reactive dyes, acid dyes and a disperse dyes. Blended fabrics of Tencel with silk and polyester give bright shades with good fastness properties^5^. It has been reported that higher colour strength (K/S) is achievable in the reactive dyeing of Tencel fabrics compared with cotton fabrics using the same amount of dye^6^.The printing/dyeing behaviors of Tencel fabrics are also reported in these work^7–11^. The dyeing bamboo and cotton fabrics with reactive dyes have the same and good wash and rubbing fastness properties. Moreover, dyed bamboo fabrics exhibited better light fastness properties than dyed cotton fabrics^12^. Urea plays an important role in the reactive printing as a reactive dye solubilizer, disaggregating agent and swelling agent for cellulose fiber during steaming process. However, use of urea poses environmental problems associated with the high nitrogen content of the printing effluent. Moreover, inorganic alkali are required in large quantities to complete the printing process, resulting in high levels of total dissolved solids in the printing effluent. Therefore, Urea/alkali reduction or elimination in reactive printing of cotton is of environmental interest. Use of biodegradable organic compounds in printing past formulation for reactive printing is a good approach of reducing the printing effluent pollution. Organic salts have been explored and found to be good alternatives to the inorganic salts in the reactive dyeing/printing of cotton fabrics^13–22^. Application and optimization parameters of trisodium nitrilotriacetate (TNA) as an organic salt in the dyeing of cotton fabrics with reactive dyes have been investigated^22^. Continuing our work^15–18^ toward cleaner production in textile industries, this study aimed to examine the use of TNA as a substitute for urea and inorganic alkali in the conventional reactive printing of cotton/cellulosic regenerated blended fabrics. A comparative study between urea and TNA in the printability of cotton/cellulosic regenerated blended fabrics with bi-functional reactive dye was thoroughly investigated. Different factors that may affect the printability of cotton/cellulosic regenerated blended fabrics, such as the concentrations of TNA, urea, dye, absence or presence of alkali and steaming time on K/S, dye fixation, dye penetration, levelling and the fastness properties were examined.

## Materials and methods

### Materials

#### Textile fibres

Textile fibres used in this study, namely bamboo (B), Tencel (T) and modal (M) fibres were purchased from SAC company from Borg Elarab, Alexandria Egypt. Egyptian cotton (Cot) fiber of type Giza 86 was also bought from Misr Spinning and Weaving Company, Elmehala Elkobra Egypt.

## Textile blended yarns

In Misr spinning and weaving company, Elmahala Elkobra, Egypt, the blow room (opening, cleaning and blending line) is a Chinese manufactured line which comprises different machine arranged in cascade form. This line consists of different machine such as Blendomat FA 009, multifunctional separator, mono-cylinder cleaner, Automixer. foreign particle separator, final cleaner and fine (intensive) cleaner. In carding process, a carding machine of type FA203 C-China) was also used.

To well be blended, the textile fibers was subjected to three drawing passages. Drawing machine of type RSB-D40-2010-China with 4/3 drafting systems were also used to accomplish this target. The combing machine is used mainly to improve the quality of the produced spun yarns by removing fiber hooks, short fibers, and any trash particles, and neps found in the processed textile fibers. In this stage of spinning, a combing machine of model Reiter E 66- 2010 was used.

Roving stage is the final process prior to the ring-spinning. The objective of this process is to draft the feed combed slivers and impart a small quantity of twists to them. During this stage, a roving machine of type FA-415 A-2003 China was used. Spinning denotes the process that converts a continuous strand of staple fibers (known as roving) into a continuous length twisted structure of fibers named for spun- yarns. The ring spinning machine of type Reiter G4-1959 was used to produce the blended spun yarns. Throughout this study, ring-spun yarns of counts 80/Ne were used as warp and weft yarns in the woven fabrics.

## Textile woven fabrics

100% Cot spun yarns, Cot/B, Cot/T and Cot/M binary blended yarns of counts 80/2/Ne were convereted into woven fabrics with a plain 1 /1 weave structure and with warp and weft densities 105 ends/inch and 75 ppi respectively. Picanol Rapier (GamMax 6- R-190) weaving machine was used to convert the blended spun yarns into woven fabrics. Lightweight, desized, scoured and bleached Cot, Cot/B, Cot/T and Cot/M fabrics with weight of 113, 111, 107 and 113.3 g/m2 respectively were used in this study.

## Dyestuffs and chemicals

**Fig. 1:**
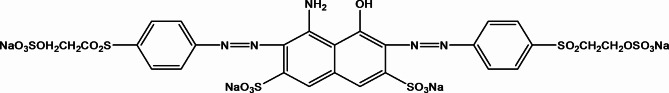
The Chemical structure of Remazol Black B (Bifunctional VS Reactive Dye).

**Fig. 2:**
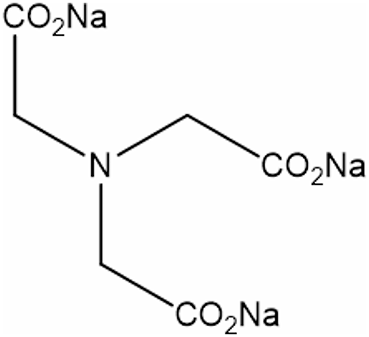
The Chemical structure of Trisodium nitrilotriacetate (TNA).

The dye used in this study was CI Reactive Black 5, bifunctional vinyl sulphone (VS) reactive dye (Remazol Black B, Fig. 1) obtained from DyStar (Egypt) and were used as received. Sodium alginate of medium viscosity, trisodium nitrilotriacetate (TNA, Fig. 2), urea, sodium bicarbonate and N, N-dimethylformamide (DMF) were of laboratory reagent grade and applied without further purification.

### Printing methods

The print pastes were prepared as follows: thickening agent, sodium alginate 36 g/Kg; dye, 10, 50 g/kg; urea or TNA, 25–150 g/kg; NaHCO_3_, 0, 25 g/kg; water, X g (up to a total of 1000 g), where X is the amount of water in grams used for 1 kg paste. The aforementioned printing pastes were applied to Cot and its blended, Cot/B, Cot/T and Cot/M fabrics according to conventional flat screen printing method^23^. Fixation of the prints was made by drying at room temperature followed by steaming for different time intervals (5–25 min) at 102^o^C.

## Colour measurements

The K/S values and colour coordinates (L*, a*, b*) of printed Cot and its blended, Cot/B, Cot/T and Cot/M fabrics were measured using an UltraScan PRO spectrophotometer (HunterLab, USA). The extent of reactive dye fixation (%F) on Cot/cellulosic regenerated blended fabrics was determined by a method used by several earlier workers^24–27^ using Eq. (1), where (K/S)_1_ and (K/S)_2_ represent the colour strength of the printed samples before and after stripping of any unfixed dye with DMF.

1 $$\%{\textrm{F}}={\frac{({\textrm{K/S}})_2}{({\textrm{K/S}})_1}}\times100$$

Reactive dye penetration (%*P*) was calculated^28^  using Eq. (2), where (K/S)_f_ and (K/S)_r_ are the colour strength values on the front and reverse of the printed fabrics respectively.

2 $$\%{\textrm{P}}={\frac{({\textrm{K/S}})_\textrm{r}}{({\textrm{K/S}})_\textrm{f}}}\times100$$

The mean color differences (∆E) on five points could represent leveling properties and penetration uniformity of bifunctional reactive dye on Cot and its blended, Cot/B, Cot/T and Cot/M fabrics. The color differences (∆E) were calculated using the measured values of CIELAB using Eq. (3)

3 $$\Delta E = [ (\Delta L*)^2+ (\Delta a*)^2+ (\Delta b*)^2]^{1/2}$$

*∆*L*, *∆*a* and *∆*b* are the difference in the color parameters of the printed fabrics.

## Fastness testing

The colour fastness of Cot and its blended fabrics were assessed according to ISO standard methods^29–32^. Fastness to washing was carried out according to *ISO 105-C06 B2S*. Fastness to an acidic and alkaline perspiration was carried out according to *ISO 105-E04*. Changing the colour of Cot and its blended fabrics and colour staining of the adjacent multi-fiber were then assessed with the ISO grey scales. Light fastness was also assessed according to *ISO105-B02* using a Xenon arc lamp test.

## Results and discussion

### Mechanical properties of textile woven fabrics

It should be noted that the characteristics of the produced blended woven fabrics are evaluated under standard conditions, namely the temperature of 20^o^C ± 2 and relative humidity of 65% ± 2, and according to different standard testing methods. Tensile properties of woven fabrics were evaluated using Tinius Olson 5CT Testing machine in accordance with ASTM D5035-11(2019)^33^. This measuring device is based upon the principle of a constant rate of extension (CRE). The test speed was set at 300 mm/min, and the gauge length was also set at 75 mm. According to the test method, the fabric sample was cut into 150 mm length and 50 mm width. Each test specimen was subjected to a uniform pre-tension of less than 0.5% of the full-scale force. For each specimen, ten readings in the weft direction and their average were obtained and calculated. For each specimen, the ultimate breaking force, breaking elongation were obtained.

Air permeability of the woven fabrics using was measured using Air permeability FX 3300 (Textest AG, Switzerland) in accordance with the standard test method ASTM D737-18^34^. For each sample, air permeability was measured at different ten positions across the test specimen surface. The average of the ten readings was also calculated. Drape is the term used to describe the way a fabric hangs under its own weight. It is one of the most important fabric characteristics since it shows how good a garment looks in use. In general, the fabric drape depends mainly on its shear and bending properties. Fabric drapeability is measured according to the British standard test method - BS-5058-1973^35^.

Table 1 shows some mechanical properties of Cot fabric and its blends with B, T and M textile fibers. The statistical analysis revealed that these properties differ significantly from one fabric to another. It was found that the tensile strength of woven Cot fabric is more than that of other fabrics. The statistical analysis proved that the tensile strength of 100% Cot fabric is more than their counterparts of binary blended ones. It is also confirmed that the tensile strength of the binary blended fabric differ from each other with the highest values associated with Cot/B fabric – 402 N, followed by Cot/T and Cot/M fabric by about 365 N and 291 N respectively. Regarding the average values of breaking elongation, the 100% Cot fabric showed the lowest value, 12.56%, whereas Cot/T binary blended fabric exhibited the highest value, 19.6%. The tearing strength of the fabric is often used to give a reasonably direct assessment of serviceability than the tensile strength. The statistical analysis confirmed that the tearing strength of 100% Cot fabric and its blends with B, T and M fibers differ remarkably at 0.01 significance level. The statistical analysis proved that Cot/T blended fabric showed the highest value of tearing strength, 2340 g, followed by 100% Cot fabric, 2310 g. The lowest value of tearing strength was accompanied by Cot/B blended fabric by about 1220 g.

Regarding the average values of drapeability, Cot/B blended fabric exhibited the highest value by approximately 80% followed by Cot/M blended fabric by about 75% and 100% Cot fabric, 74%. The Cot/T blended fabric gave the lowest value of drapeability by about 70%. Regarding average values of air permeability, Cot/T blended fabrics are associated with the highest value, 132 cm3/cm2.sec, followed by 100% Cot fabric by about 123 cm3/cm2.sec. The Cot/B and Cot/M blended fabrics exhibited the lowest and equal air permeability values i.e. 96 cm3/cm2.sec.

**Table 1:** Mechanical properties of textile woven fabrics.

Fabrics	Mechanical properties				
	Tensile strength (Newton)	Breaking elongation (%)	Tearing strength (gm)	Drapeability (%)	Air permeability (cm3/cm2.sec)
Cot	472 (480)*	12.5 (11.9)	2310 (2323)	74 (72)	123 (115)
Cot/B	402 (413)	17.2 (16.8)	1220 (1225)	80 (74)	96 (90)
Cot/T	365 (378)	19.6 (18.8)	2340 (2348)	70 (66)	132 (124)
Cot/M	291 (309)	16.1 (15.7)	1600 (1610)	75 (72)	96 (91)

*The values in parenthesis concern the mechanical properties of the printed fabrics.

Table 1 introduces the mechanical properties of grey and printed woven fabrics. From this table, it is clear that there are insignificant differences between the two types of woven fabrics regarding their mechanical properties. Compared to grey woven fabrics, the tensile strength, breaking elongation, and tearing strength of the printed fabric samples are increased with insignificant values. On the other hand, the air permeability and drapeablity of the printed fabrics were decreased with insignificant values in comparison with blank samples.

### Effect of TNA and urea concentration on reactive printing of cot and its blended fabrics

Blending the functional fibers such as B, T and M with natural Cot fiber will open new opportunities for the development of the textile end products. It is of interest to substitute urea with TNA in reactive printing of Cot fabrics. Recently, we have explored the viability of using TNA in the reactive dyeing of Cot fabric as an exhausting and fixing agent^36^. Therefore, it is anticipated that its participating in reactive printing of Cot/cellulosic regenerated blended fabrics under investigation would be favorable. To evaluate the feasibility of using TNA in the reactive printing of Cot/cellulosic regenerated blended fabrics, printing was conducted using bifunctional VS reactive dye in a comparative manner between using TNA and urea in the presence or absence of alkali. Tables 2 and 3 show the effect of using three print pastes (urea/25 g/Kg NaHCO_3_, TNA/25 g/Kg NaHCO_3_ and TNA/without NaHCO_3_) at different concentrations of urea and TNA on K/S and %F values of printed Cot and its blended, Cot/B, Cot/T and Cot/M fabrics using Remazol Black B. It can be clearly seen from the results that the K/S and %F of the printed Cot and its blended fabrics using TNA/25 g/Kg NaHCO_3_ and TNA/without NaHCO_3_ print pastes reveal the highest K/S and %F values. All samples printed using TNA print paste show the highest K/S and %F values compared with those obtained using TNA/25 g/Kg NaHCO_3_. As the trend goes from TNA > TNA/25 g/Kg NaHCO_3_ > urea/25 g/Kg NaHCO_3_, this could be attributed to the moderate basicity of TNA and its capacity of buffering action in the print paste. Consideration of the K/S and %F values obtained (Tables 2 and 3) for Remazol Black B relative TNA concentrations would lead to the optimum conditions of TNA and NaHCO_3_ for better results are (50 g/Kg TNA + 25 g/Kg NaHCO_3_ and 75 g/Kg TNA/without NaHCO_3_). Theses optimum conditions were used for the subsequent study. Also printed Cot/M blended fabrics exhibited higher K/S and %F values compared with Cot, Cot/B and Cot/T fabrics. In other words, K/S values of printed Cot/M blended fabrics using three printing pastes TNA, TNA/C and U/C are 30.83, 29.36 and 29.02 respectively. Also, F% values of printed Cot/M blended fabrics using three printing pastes TNA, TNA/C and U/C are 98.1, 97.8 and 97.7 respectively. The highest K/S and F% values are observed using TNA print past. Therefore, printing of Cot and Cot/M blended fabrics with VS reactive dyes using TNA print past could be recommended for industrial application. Moreover, selected reactive dye structures will be designed and synthesized in future work to study reactive printing behavior of Cot/M blended fabric and the outcome of this work will be published in another publication.

**Table 2:** K/S values of printed cot and its blended Cot/B, Cot/T and Cot/M fabrics using bifunctional VS reactive dye (50 g/kg) in different print pastes at different concentrations of urea and TNA with and without using 25 g/kg NaHCO_3_.

Conc^a^ [g/l]	K/S after DMF^b^											
	U/C				TNA/C				TNA			
	Cot	Cot/B	Cot/T	Cot/M	Cot	Cot/B	Cot/T	Cot/M	Cot	Cot/B	Cot/T	Cot/M
25	27.15	27.65	27.63	28.17	20.37	18.88	19.30	21.72	15.02	13.74	14.43	16.42
50	27.23	27.87	27.67	28.38	28.17	28.47	29.36	29.51	28.99	28.83	28.32	29.62
75	27.37	27.95	27.94	28.56	28.01	27.85	28.13	28.57	29.40	29.02	29.87	30.83
100	27.44	28.18	28.15	29.02	27.86	26.03	26.18	27.76	28.58	28.31	28.04	29.96
125	27.24	27.58	27.67	28.35	26.70	25.55	25.63	25.72	27.45	26.67	27.42	28.79
150	27.10	26.42	27.42	28.17	26.45	24.67	24.38	24.86	27.14	25.88	26.11	27.34

^a^Print paste was made from a constant amount of 36 g/kg sodium alginate and the prints obtained were steamed at 102^o^C for 15 min.

^b^U/C, urea in the presence of 25 g/kg NaHCO_3_; TNA/C, Trisodium nitrilotriacetate in the presence of 25 g/kg NaHCO_3_; TNA, Trisodium nitrilotriacetate without NaHCO_3_.

**Table 3:** Fixation values of printed cot and its blended Cot/B, Cot/T and Cot/M fabrics using bifunctional VS reactive dye (50 g/kg) in different print pastes at different concentrations of urea and TNA with and without using 25 g/kg NaHCO_3_.

Conc [g/l]	% F											
	U/C				TNA/C				TNA			
	Cot	Cot/B	Cot/T	Cot/M	Cot	Cot/B	Cot/T	Cot/M	Cot	Cot/B	Cot/T	Cot/M
25	93.8	94.5	94.5	96.7	85.5	87.3	84.8	87.7	82.8	85.6	81.9	83.2
50	94.1	94.8	94.7	96.8	96.8	97.2	97.7	97.8	97.3	97.2	97.4	97.8
75	94.6	94.8	94.8	97.1	96.0	96.3	96.9	97.3	97.9	97.8	98.0	98.1
100	95.6	95.5	95.4	97.7	95.6	95.8	95.7	96.3	97.2	97.0	97.3	97.5
125	94.5	94.7	94.6	97.0	95.0	95.3	95.2	95.8	96.6	96.3	97.2	97.0
150	93.7	93.5	94.5	96.8	94.7	94.8	94.7	95.2	96.3	96.6	97.0	96.8

**Fig. 3:**
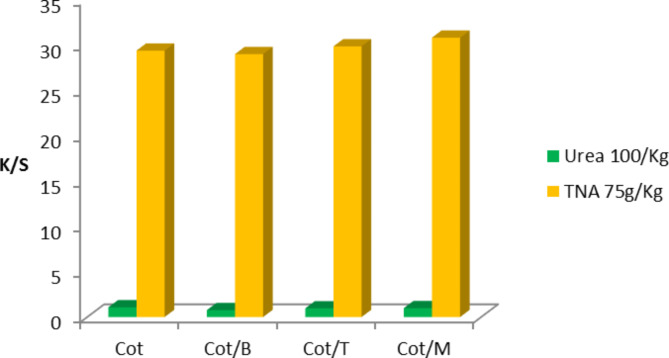
K/S values of DMF-extracted printed Cot and its blended Cot/B, Cot/T and Cot/M fabrics using bifunctional VS reactive dye (50 g/kg) at the selected urea and TNA concentrations in the absence of NaHCO_3_ (prints obtained after steaming at 102^o^C for 15 min).

Figure 3. describes the effect of alkali absence on K/S values of DMF-extracted printed Cot and its blended Cot/B, Cot/T and Cot/M fabrics using bifunctional VS reactive dye (50 g/kg) at the obtained optimum concentrations of urea and TNA (Tables 2 and 3). From which it is clear that, using urea 100 g/kg/without alkali has very low K/S values on printed samples compared with those samples using TNA 75 g/Kg/without alkali. In addition, the use of TNA can not only be used as a complete substitution of urea, but also suggests reactive printing without alkali, which are in favor for the reactive printing of Cot fabrics. A similar result using TNA as exhausting and fixing agent in reactive dyeing of Cot fabrics has recently been reported^36^.

### Effect of steaming fixation time on reactive printing of cot and its blended fabrics

**Fig. 4:**
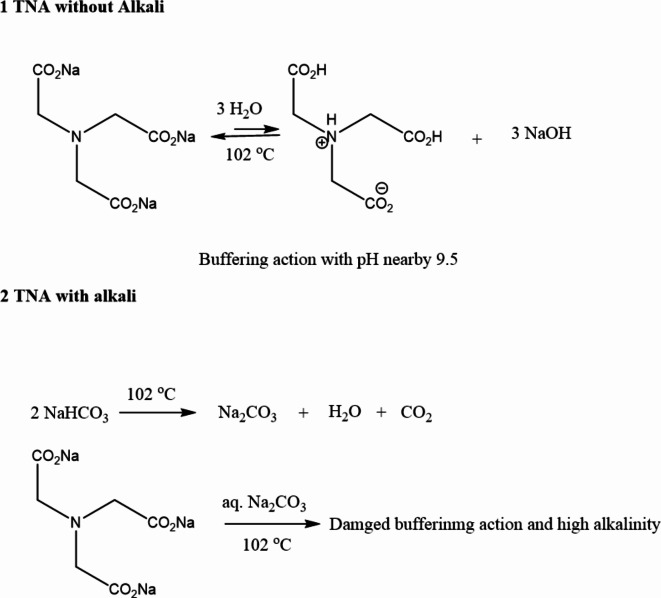
Effect of steaming at 102^o^C on the TNA in the presence and absence of NaHCO_3_.

**Fig. 5:**
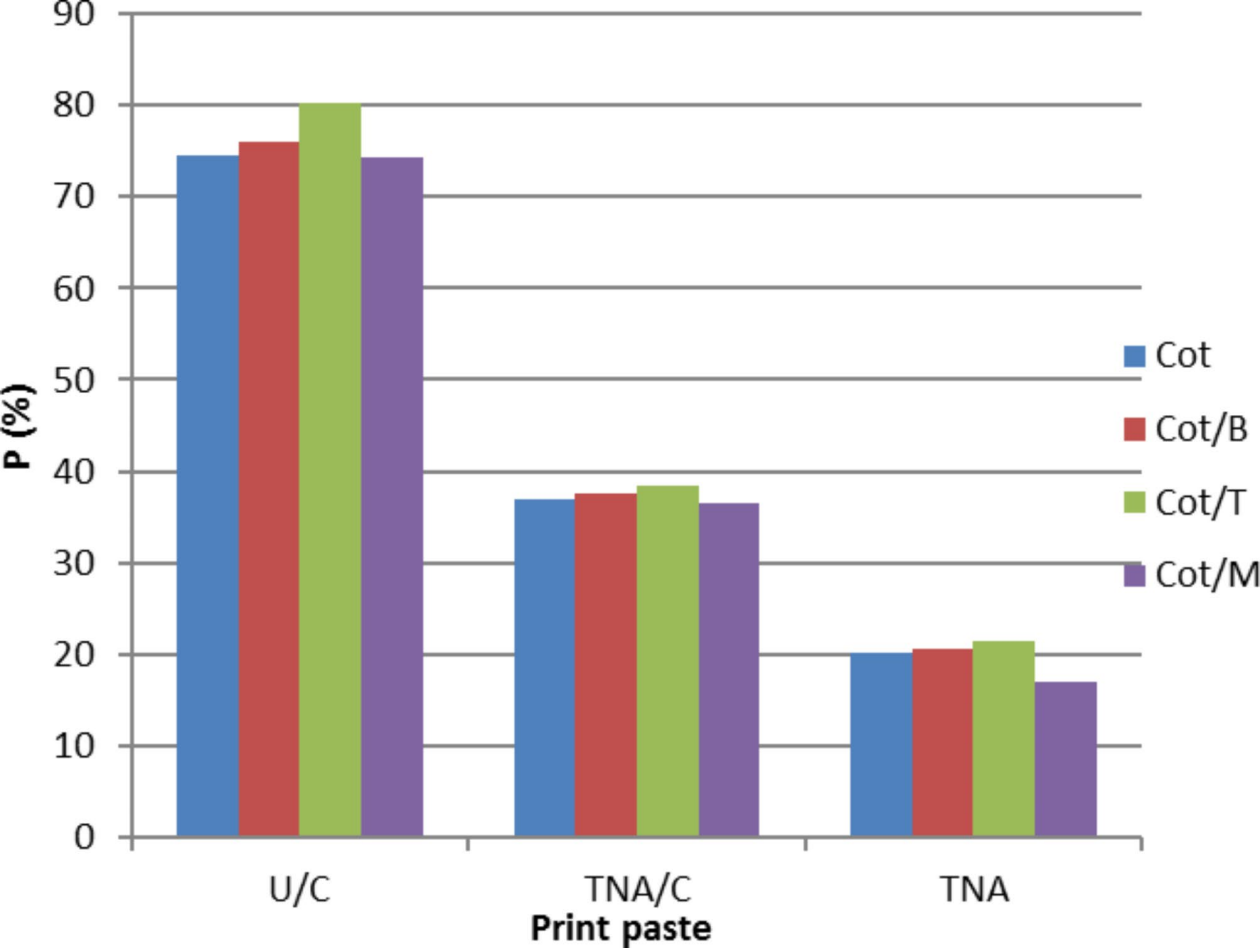
Per cent penetration of bifunctional VS reactive dye (10 g/kg) into the printed Cot and its blended Cot/B, Cot/T and Cot/M fabrics using U/C, TNA/C and TNA print pastes. Prints obtained after steaming at 102^o^C for 7.5 min.

Tables 4 and 5 show the effect of steaming fixation time, at 102 °C for 5–25 min, on the K/S and F% values on Cot and its blended fabrics printed with bifunctional VS reactive dye (10 g/kg). Compared with U/C and TNA/C, the data obtained using TNA only were generally higher confirming further the usefulness of TNA as both fixing and dye-uptake agent in accordance with our reported results of reactive dyeing^36^. However, the K/S and F% values of the printed fabrics using TNA/C print pastes were decreased dramatically by increasing the steaming time from 5 to 25 min. This result reflects the negative effect of mixing sodium bicarbonate with TNA in the print paste formulation as it affects both the VS reactive dye uptake and TNA buffering action as well. A tentative mechanism shown in Fig. 4. reveals an explanation for this result. It is known that NaHCO_3_ is thermally decomposed to Na_2_CO_3_, water, and CO_2_. Therefore, as the streaming time increases, the accumulation of Na_2_CO_3_ increases, which increases the alkalinity of the print paste and surface negative charge of the cellulosic fabrics, thus hindering further dye uptake and increasing the dye hydrolysis as reflected by the color strength and dye fixation valuers, respectively. On the other hand, previous results on reactive dyeing of Cot with TNA indicated that aqueous TNA as an organic slat base with pH 9.5 performed well as an exhausting and fixing agent of reactive dyeing^36^. Thus, it is expected that adding alkali to TNA in the print paste and at high temperatures would damage its buffering action and increase the basicity of the medium (Fig. 5.). It is noteworthy to mention the unique effect of TNA as fixing agent and dye uptake without the need of alkali in the print paste.

**Table 4:** K/S values of printed cot, Cot/B, Cot/T and Cot/M fabrics using bifunctional VS reactive dye (10 g/kg) in three print pastes at different steaming times.

Steaming time (min)	K/S after DMF^a^											
	U/C				TNA/C				TNA			
	Cot	Cot/B	Cot/T	Cot/M	Cot	Cot/B	Cot/T	Cot/M	Cot	Cot/B	Cot/T	Cot/M
5	15.37	14.90	15.07	15.43	12.36	11.38	12.57	12.45	15.54	14.96	15.19	15.56
10	14.86	14.39	14.51	15.20	11.31	10.86	11.27	11.82	15.62	15,47	15.70	15.83
15	14.71	14.21	14.28	14.93	8.17	7.86	8.06	8.50	16.19	15.91	16.07	16.43
20	14.36	13.71	13.88	14.75	5.47	4.83	5.14	5.63	16.47	16.04	16.25	16.74
25	13.45	12.81	13.42	13.85	2.65	2.43	2.51	2.76	16.72	16.37	16.58	16.95

^a^Three print pastes (100 g/kg urea + 25 g/Kg NaHCO_3_, 50 g/Kg TNA + 25 g/Kg NaHCO_3_ and 75 g/Kg TNA without NaHCO_3_) were selected for this study.

**Table 5:** Fixation values of printed cot, Cot/B, Cot/T and Cot/M fabrics using bifunctional VS reactive dye (10 g/kg) in three print pastes at different steaming times.

Steaming time (min)	% F											
	U/C				TNA/C				TNA			
	Cot	Cot/B	Cot/T	Cot/M	Cot	Cot/B	Cot/T	Cot/M	Cot	Cot/B	Cot/T	Cot/M
**5**	96.2	96.0	96.1	96.3	95.7	95.6	95.6	95.8	96.5	96.4	96.5	96.7
**10**	96.7	96.4	96.6	96.9	95.4	95.2	95.3	95.5	96.8	96.6	96.7	96.1
**15**	97.3	96.9	97.1	97.5	95.0	94.7	94.8	95.2	97.4	96.8	96.9	79.4
**20**	96.9	96.5	96.7	97.00	94.8	93.4	93.5	93.9	97.5	97.2	97.4	97.7
**25**	96.0	95.7	95.9	96.4	93.2	93.0	93.1	93.6	97.7	97.4	97.6	98.0

### Reactive dye penetration and levelling properties

**Fig. 6:**
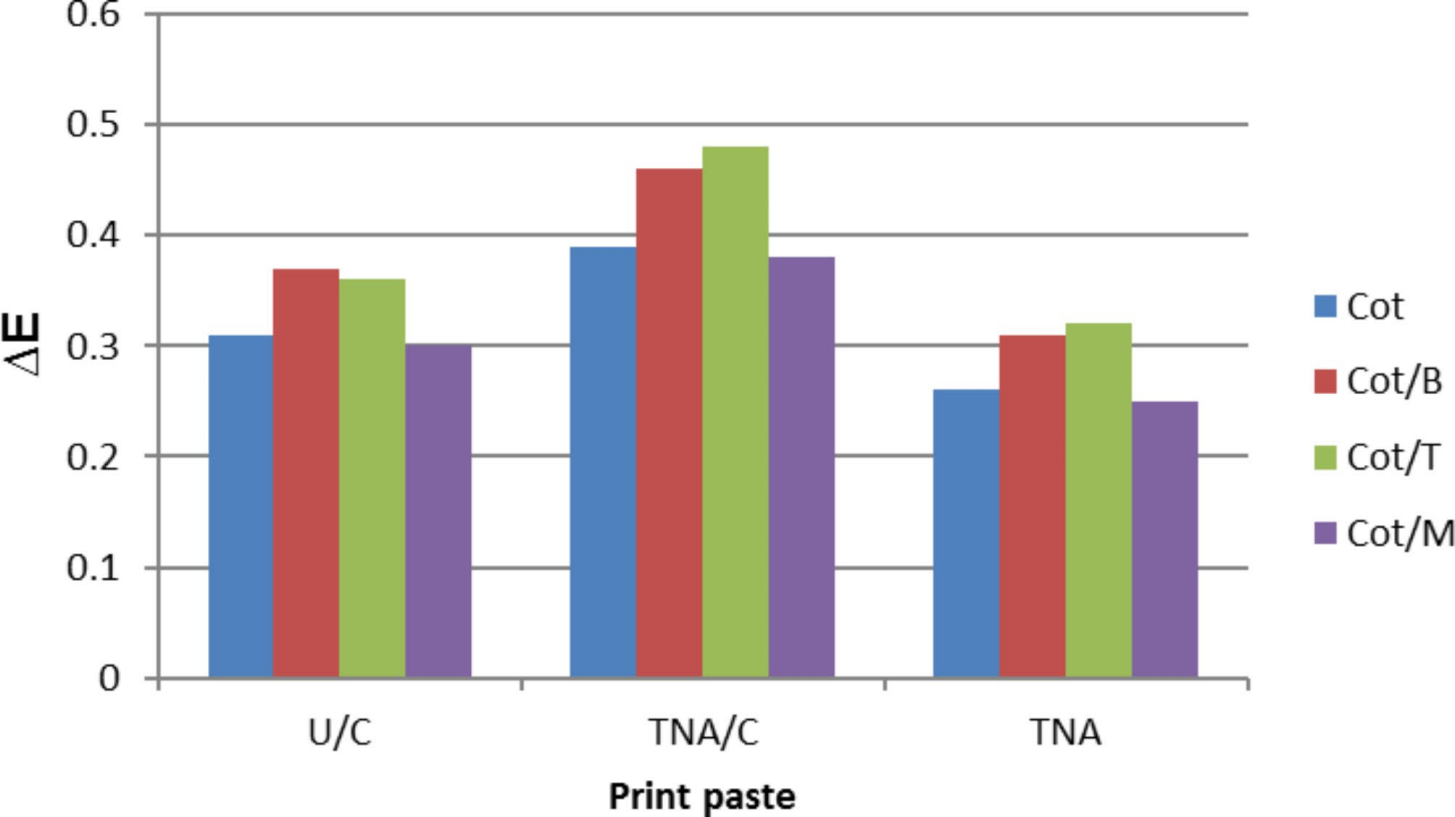
Average colour differences (∆E) of printed Cot and its blended Cot/B, Cot/T and Cot/M fabrics using different print pastes with bifunctional VS reactive dye (10 g/kg). Prints obtained after steaming at 102^o^C for 15 min.

Figure 6. describes penetration values of bifunctional VS reactive dye (10 g/kg) into Cot and its blended Cot/B, Cot/T and Cot/M fabrics using selected print pastes (100 g/kg urea + 25 g/Kg NaHCO_3_, 50 g/Kg TNA + 25 g/Kg NaHCO_3_ and 75 g/Kg TNA without NaHCO_3_). The results illustrated in Fig. 4 are the average ones for two runs made at 5 and 10 min steaming times. The result indicates that TNA/C and TNA printing shows the best results compared with U/C conventional printing. Also printed Cot/M fabrics exhibited lower penetration % values than those of Cot, Cot/B and Cot/T fabrics. This confirm the previous results in Table 1 that, printed Cot/M fabrics had higher K/S values than those of Cot, Cot/B and Cot/T fabrics in all cases. This work is a step forward for overcoming the challenge to print the lightweight fabrics with minimum penetration%.

The levelling properties (∆E values) of Cot and its blended Cot/B, Cot/T and Cot/M fabrics printed with bifunctional VS reactive dye (10 g/Kg) using the selected print pastes mentioned above were shown in Fig. 6, from which, it was found that the printed fabrics show good levelling properties in all cases. When TNA was used, all the average ∆E values of printed fabrics were found within 0.25–0.31 range. Also, when U/C was used, all the average ∆E values of printed fabrics were found within 0.30–0.37 range. Finally, when TNA/C was used, all the average ∆E values of printed fabrics were found within 0.38–0.49 range. Therefore, the printed fabrics obtained by TNA had the best leveling properties relative to those obtained by TNA/C and U/C. In addition, among the printed fabrics using U/C, TNA/C and TNA print pastes, the order of levelness found was: Cot/M > Cot > Cot/B > Cot/T.

### Color and fastness properties

Table 6 shows the values obtained for the colour coordinates (L*, a*, b*) of printed Cot and its blended Cot/B, Cot/T and Cot/M fabrics with bifunctional VS reactive dye. L* represents the lightness coordinate. a* represents the redness/greenness coordinate, with + a* indicating red, and -a* indicating green. b* represents the yellowness/blueness coordinate, with + b* indicating yellow, and -b* indicating blue. It is clear from Table 6 that, the printed fabrics using TNA printing paste exhibited higher dye uptake than those using U/C and TNA/C printing pastes, as evidenced by a decrease in L* values. Also printed Cot/M fabrics exhibited higher dye uptake than those Cot, Cot/B and Cot/T fabrics as evidenced by a decrease in L* values using three print pastes. It was observed also that, all the color coordinates of printed Cot and its blended Cot/B, Cot/T and Cot/M fabrics were positive with respect to red/green a* and negative with respect to yellow/blue b* coordinates depending on the uptake of bifunctional VS reactive dye used; therefore, all of them lie in the blue-red quadrant of the color space diagram. The results also indicated that color coordinate CIE L*, a*, and b* values of printed Cot/B, Cot/T and Cot/M blended fabrics are nearly at the same chromaticity zone compared with those of the printed Cot fabrics using three printing pastes.

**Fig. 7:**
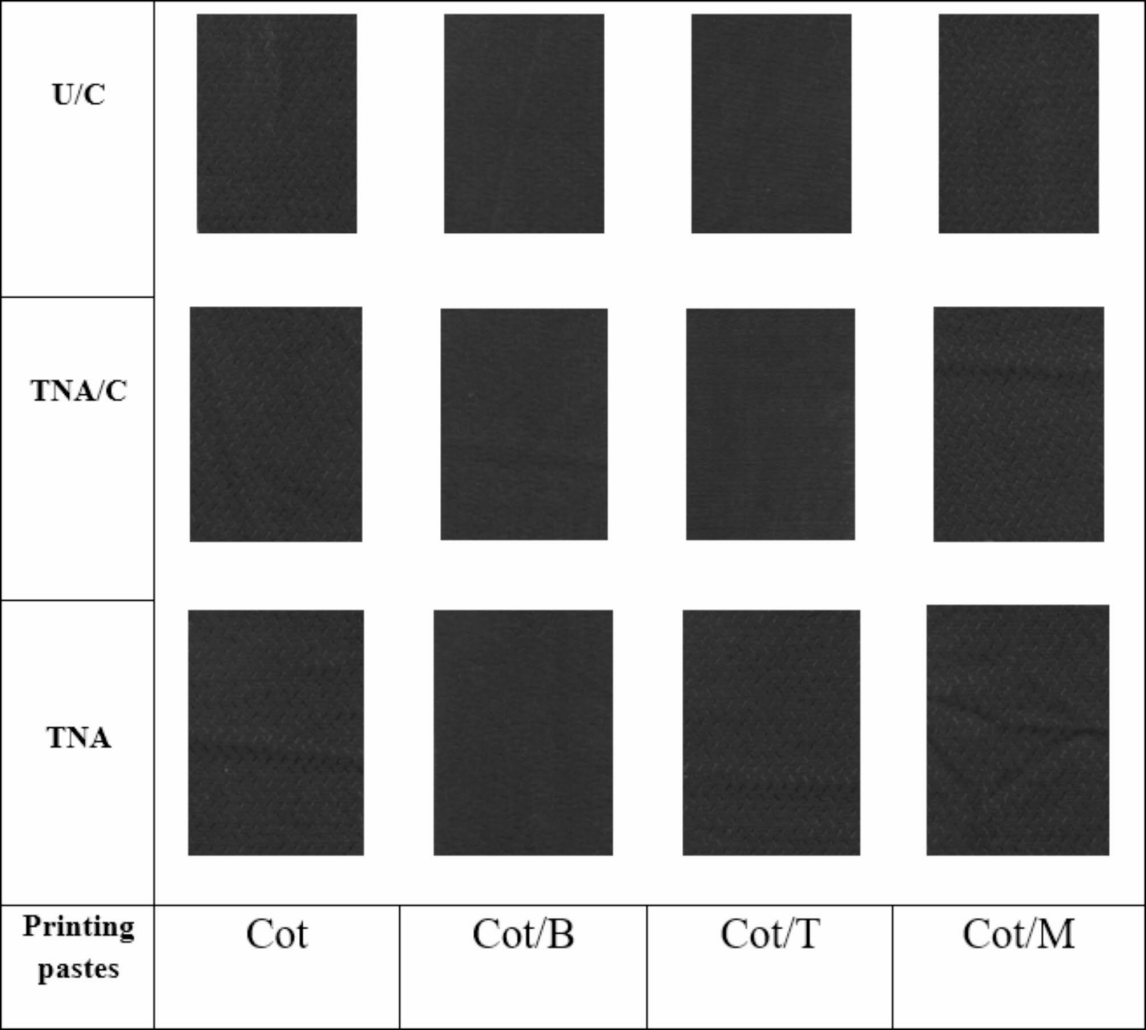
Images of printed Cot and its blended Cot/B, Cot/T and Cot/M fabrics using three print pastes (100 g/kg urea + 25 g/Kg NaHCO_3_, 50 g/Kg TNA + 25 g/Kg NaHCO_3_ and 75 g/Kg TNA without NaHCO_3_) and bifunctional VS reactive dye (50 g/kg).

Images of printed Cot and its blended Cot/B, Cot/T and Cot/M fabrics using three print pastes (100 g/kg urea + 25 g/Kg NaHCO_3_, 50 g/Kg TNA + 25 g/Kg NaHCO_3_ and 75 g/Kg TNA without NaHCO_3_) and bifunctional VS reactive dye (50 g/kg), as illustrated in Fig. 7.

**Table 6:** Colorimetric data of printed cot and its blended Cot/B, Cot/T and Cot/M fabrics using bifunctional VS reactive dye (50 g/kg).

Printing pastes ^a^	Cot			Cot/B			Cot/T			Cot/M		
	L*	a*	b*	L*	a*	b*	L*	a*	b*	L*	a*	b*
U/C	15.82	0.68	-6.03	17.10	0,01	-6.41	15.99	0.20	-6.23	15.01	0.10	-5.23
TNA/C	16.10	0.13	-4.94	16.62	0.26	-6.16	16.23	1.17	-5.60	16.08	0.69	-6.27
TNA	14.67	0.97	-4.77	15.62	0.36	-4.61	14.36	1.04	-2.77	14.25	0.74	-3.44

^a^Three print pastes (100 g/kg urea + 25 g/Kg NaHCO_3_, 50 g/Kg TNA + 25 g/Kg NaHCO_3_ and 75 g/Kg TNA without NaHCO_3_) were selected for this study.

The rubbing, washing and perspiration fastness results of Cot and its blended Cot/B, Cot/T and Cot/M fabrics printed with bifunctional VS reactive dye using the three print pastes (U/NaHCO3, TNA/NaHCO3 and TNA) are listed in Table 7. The results obtained are excellent to good for three print pastes.

**Table 7:** Fastness properties of printed cot and its blended Cot/B, Cot/T and Cot/M fabrics using bifunctional VS reactive dye (50 g/kg)*.

Printed fabrics	Printing pastes^a^	Fastness to rubbing		Washing fastness^b^			Perspiration fastness					
							Acidic			Alkaline		
		Dry	Wet	Alt	SC	SW	Alt.	SC	SW	Alt.	SC	SW
**Cot**	**U/C**	4–5	3–4	5	5	5	5	5	5	5	5	5
	**TNA/C**	4–5	3–4	5	5	5	5	5	5	5	5	5
	**TNA**	4–5	3–4	5	5	5	5	5	5	5	5	5
**Cot/B**	**U/C**	4–5	4	5	5	5	5	5	5	5	5	5
	**TNA/C**	4	3–4	5	5	5	5	5	5	5	5	5
	**TNA**	4–5	4	5	5	5	5	5	5	5	5	5
**Cot/T**	**U/C**	4–5	3–4	5	5	5	5	5	5	5	5	5
	**TNA/C**	4	3–4	5	5	5	5	5	5	5	5	5
	**TNA**	4–5	4	5	5	5	5	5	5	5	5	5
**Cot/M**	**U/C**	4–5	3–4	5	5	5	5	5	5	5	5	5
	**TNA/C**	4	3–4	5	5	5	5	5	5	5	5	5
	**TNA**	4–5	4	5	5	5	5	5	5	5	5	5

*The prints obtained were steamed at 102^o^ C for 15 min.

^a^U/C, urea/NaHCO_3_; TNA/C, Trisodium nitrilotriacetate/NaHCO_3_.

^b^Alt, alteration; SC, staining on cotton; SW, staining on wool.

## Conclusion

From the study of blending Cot fibre with regenerated cellulosic fibres and converted into lightweight woven fabrics, it was found that the mechanical properties of such blended fabrics (air permeability, traceability, tearing strength, breaking elongation and tensile strength) were very good and sometimes better than those of Cot fabrics. The viability of using TNA as an alternative to urea in reactive dye print pastes for such blended fabrics is explored. The results revealed that TNA/without alkali printing shows the best results compared with urea/alkali conventional printing. The color fastness rating for washing, perspiration, and rubbing the printed samples was excellent to good. We believe this work is a step forward in overcoming the challenge of printing lightweight fabrics with minimum penetration and cleaner production within the textile industry. A further study of blending the functional fibres with traditional ones, whether artificial or natural, would be of value for urea/alkali-free printing of cellulosic blended fabrics using selected reactive dye structures, and the outcome of this study will be published in another publication.

## Data Availability

All data generated or analyzed during this study are included in this published article.
